# Dihydromyricetin Ameliorates Inflammation-Induced Insulin Resistance via Phospholipase C-CaMKK-AMPK Signal Pathway

**DOI:** 10.1155/2021/8542809

**Published:** 2021-10-05

**Authors:** Lianjie Hou, Fangyi Jiang, Bo Huang, Weijie Zheng, Yufei Jiang, Gengyuan Cai, Dewu Liu, Ching Yuan Hu, Chong Wang

**Affiliations:** ^1^Guangdong Provincial Key Lab of Agro-Animal Genomics and Molecular Breeding, National Engineering Research Center for Breeding Swine Industry, College of Animal Science, South China Agricultural University, China; ^2^The Sixth Affiliated Hospital of Guangzhou Medical University, Qingyuan City People's Hospital, Qingyuan, 511518 Guangdong, China; ^3^Department of Human Nutrition, Food and Animal Sciences, College of Tropical Agriculture and Human Resources, University of Hawaii at Manoa, USA

## Abstract

Patients with metabolic syndrome have a higher risk of type II diabetes and cardiovascular disease. The metabolic syndrome has become an urgent public health problem. Insulin resistance is the common pathophysiological basis of metabolic syndrome. The higher incidence of insulin resistance in obese groups is due to increased levels of inflammatory factors during obesity. Therefore, developing a therapeutic strategy for insulin resistance has great significance for the treatment of the metabolic syndrome. Dihydromyricetin, as a bioactive polyphenol, has been used for anti-inflammatory, antitumor, and improving insulin sensitivity. However, the target of DHM and molecular mechanism of DHM for preventing inflammation-induced insulin resistance is still unclear. In this study, we first confirmed the role of dihydromyricetin in inflammation-induced insulin resistance in vivo and in vitro. Then, we demonstrated that dihydromyricetin resisted inflammation-induced insulin resistance by activating Ca^2+^-CaMKK-AMPK using signal pathway blockers, Ca^2+^ probes, and immunofluorescence. Finally, we clarified that dihydromyricetin activated Ca^2+^-CaMKK-AMPK signaling pathway by interacting with the phospholipase C (PLC), its target protein, using drug affinity responsive target stability (DARTS) assay. Our results not only demonstrated that dihydromyricetin resisted inflammation-induced insulin resistance via the PLC-CaMKK-AMPK signal pathway but also discovered that the target protein of dihydromyricetin is the PLC. Our results provided experimental data for the development of dihydromyricetin as a functional food and new therapeutic strategies for treating or preventing PLC.

## 1. Background

With the changes in modern lifestyles, such as excessive energy intake, lack of regular exercise, and accelerated life rhythm, the incidence of metabolic syndrome-related diseases has increased year by year [1]. Metabolic syndrome is defined as a disorder of energy use and storage. This syndrome is characterized by central obesity, dyslipidemia, raised blood pressure, and high blood glucose levels [2]. Although the prevalence of metabolic syndrome varies globally based on race, age, and gender, the incidence rate is as high as 10-84%. Patients with metabolic syndrome have a higher risk of type II diabetes and cardiovascular disease [3]. Therefore, the metabolic syndrome has become an urgent public health problem.

Insulin resistance is the common pathophysiological basis of metabolic syndrome [4]. Insulin resistance is defined as a decrease in insulin sensitivity in the major insulin target organs such as adipose tissue, liver, and muscle. Insulin resistance is one of the significant characteristics of type II diabetes [5]. Studies have shown that insulin resistance has become a high-risk factor for chronic diseases such as type II diabetes, nonalcoholic fatty liver disease, cardiovascular disease, and even Alzheimer's disease [6]. Therefore, developing a therapeutic strategy for insulin resistance has great significance for the treatment of the metabolic syndrome.

The increase of proinflammatory cytokines, such as IL-1*β*, IL-6, TNF*α*, and MCP1, in adipose tissue during obesity, is the direct cause of insulin resistance [7]. Among the proinflammatory cytokines that induce insulin resistance, tumor necrosis factor *α* (TNF*α*) is the most representative one. Inflammatory cytokines impair insulin signaling and trigger insulin resistance. The main pathways of inflammation-induced insulin resistance include nuclear factor-kappa B (NF-*κ*B) signaling pathway and c-Jun NH2-terminal kinase (JNK) signaling pathway [8]. NF-*κ*B inhibits the activation of phosphatidylinositol 3-kinase- (PI3K-) protein kinase B (AKT) signaling pathway by inhibiting phosphorylation of insulin receptor substrate (IRS), resulting in insulin resistance [9]. JNK inhibits the PI3K-AKT signaling pathway by downregulating the expression and phosphorylation of the IRS, resulting in insulin resistance [10]. Therefore, inhibiting chronic inflammation is a promising therapeutic strategy to improve insulin resistance.

Dihydromyricetin (DHM), also known as ampelopsin, is the main bioactive polyphenol in rattan tea. Rattan tea has been used for anti-inflammatory in China and other Asian countries for several centuries [11]. DHM exerted its anti-inflammatory effect in rats through suppressing NF-*κ*B signaling in macrophage [12]. DHM concentration dependently increased the glucose uptake in insulin-resistant 3T3-L1 adipocytes induced by dexamethasone [13]. DHM also improved skeletal muscle insulin sensitivity by partially inducing autophagy via activation of the AMPK-PGC-1 alpha-Sirt3 signaling pathway [14]. DHM attenuated metabolic syndrome and improves insulin sensitivity by upregulating phosphorylation of IRS in db/db mice [15]. Currently, most DHM-related research reported that DHM functions depend on its activation of AMPK; but AMPK is not the direct target of DHM in cells, and the target of DHM in cells is still unclear. To our knowledge, there is no published paper demonstrated the DHM effect on inflammation-induced insulin resistance.

In this study, we used high-fat diet (HFD) feeding to induce insulin resistance in vivo. Gavage DHM was used to determine DHM function in reducing the level of inflammation and inhibiting insulin resistance in obese mice. 3T3-L1 cells were treated with TNF*α* as a model of inflammation-induced insulin resistance in vitro. We have elucidated the molecular mechanism of DHM for preventing inflammation-induced insulin resistance in 3T3-L1 using immunofluorescence, signal pathway blocking, and drug affinity responsive target stability (DARTS) assay. Our results provided not only experimental data for the development of DHM as a functional food additive but also offered a new therapeutic strategy for treating insulin resistance.

## 2. Materials and Methods

### 2.1. Animal Experiment

Thirty-six 18-day-old specific pathogen free (SPF) healthy male C57B/L6 mice were purchased from the Animal Experiment Center of Guangdong Province. The mice were housed under a 12 h light and 12 h dark cycle (7 am and 7 pm, 25°C, and 70~80% humidity). The mice were divided into three groups (*n* = 12) randomly: chow diet+PBS gavage (control), the HFD+PBS gavage (HFD), and the HFD+DHM gavage (DHM). Two-hundred microliters of DHM (200 mg/kg body weight) was administered orally by gavage to the DHM group daily, while the control group and the HFD group were administered the same volume of PBS each day. We choose the rational dose of DHM (200 mg/kg body weight) according to the literature [16, 17]. The BW and feed intake of the mice were recorded every week. At week 10, we analyzed the body composition of mice using a nuclear magnetic resonance system. At week 11, we tested insulin sensitivity in mice using an oral glucose tolerance test. At week 12, the mice were euthanized to collect serum, inguinal white adipose tissue (iWAT), epididymis white adipose tissue (eWAT), and other tissues for further analysis. All experiments were conducted following “The Instructive Notions with Respect to Caring for Laboratory Animals” issued by the Ministry of Science and Technology of the People's Republic of China. All experimental protocols and methods were approved by the College of Animal Science, South China Agricultural University.

### 2.2. Oral Glucose Tolerance Test

After fasting for about 12 hours, the blood glucose of the mice was measured by ACCU-CHEK Mobile (Roche, Mannheim, Germany). Then, the mice were given oral administration of glucose (1.0 g/kg) solution. After 30, 60, 90, 120, and 180 min, blood samples were collected via the tail vein for the measurement of blood glucose levels.

### 2.3. Hematoxylin-Eosin Staining (H&E)

The mouse adipose tissue was fixed in 4% formaldehyde (DaMao, Tianjin, China) at room temperature for 48 h. The method used for the H&E staining has been described previously [16]. The adipocyte area is calculated by the Image J software.

### 2.4. Body Composition Analysis

Fat mass, lean mass, and body composition were determined using a nuclear magnetic resonance system according to the manufacturer's instruction (Body Composition Analyzer MiniQMR23-060H-I, Niumag Corporation, Shanghai, China).

### 2.5. 3T3-L1 Culture and Inflammatory Induce 3T3-L1 Insulin Resistance

The 3T3-L1 cell line used in this study was purchased from American Type Culture Collection (ATCC, VA, USA). 3T3-L1 was cultured in DMEM/High Glucose (Hyclone, PA, USA) with 10% Fetal Bovine Serum (Gibco, NY, USA). 3T3-L1 cells were seeded in 24-well plates (4 × 10^5^/cm^2^). After 24 h, we treated the 3T3-L1 with TNF*α* (MedChemExpress, Monmouth Junction, USA) at the concentration of 1 ng/mL for 5 days to induce 3T3-L1 insulin resistance. We treated 3T3-L1 cells with TNF*α* and DHM (1 *μ*M, Sigma Chemical Inc., Louis, MO, USA) for 5 days to demonstrate DHM ameliorated inflammation-induced insulin resistance. We treated 3T3-L1 cells with TNF*α*, DHM, and Compound C (the AMPK inhibitor, 5 *μ*M, MedChemExpress, Monmouth Junction, USA) for 5 days to demonstrate DHM ameliorated inflammation-induced insulin resistance through AMPK. We treated 3T3-L1 cells with TNF*α*, DHM, and STO-609 (the CaMKK inhibitor, 10 ng/mL, MedChemExpress, Monmouth Junction, USA) for 5 days to demonstrate DHM ameliorated inflammation-induced insulin resistance through CaMKK. We treated 3T3-L1 cells with TNF*α*, DHM, and U73122 (1 *μ*M, MedChemExpress, Monmouth Junction, USA) for 5 days to demonstrate DHM ameliorated inflammation-induced insulin resistance through the PLC-IP3 receptor pathway.

### 2.6. Glucose Uptake Assay

Five days after treatments, the glucose uptake was assayed by 2-NBDG (MedChemExpress, Monmouth Junction, USA) according to the manufacturer's protocol. In brief, 3T3-L1 were incubated with or without media containing 10 nM insulin (Sigma Chemical Inc., Louis, MO, USA) for 10 minutes. The media were changed to low-glucose DMEM containing 150 *μ*g/mL 2-NBDG for 60 minutes at 37°C. The medium was removed, and cells were washed 5 times with PBS. Nikon Eclipse Ti-s microscopy (Nikon, Tokyo, Japan) was used to observe fluorescence. Fluorescent data were acquired at excitation and emission wavelengths of 490 and intensity at 525 nm.

### 2.7. Methyl Thiazolyl Diphenyl-Tetrazolium Bromide (MTT)

3T3-L1 cells were seeded in 96-well plates at a density of 3 × 10^4^/cm^2^. After 12 h culture, 15 *μ*L of treatments (TNF or DHM) with different concentrations was added to the cells for another 24 h of incubation. MTT was performed according to the manufacturer's protocol (M1020-500T, Solarbio, Beijing, China).

### 2.8. Calcium (Ca^2+^) Imaging

Ca^2+^ was measured by a Ca^2+^ fluorescent probe fluo-4-AM kit following the manufacturer's instructions. 3T3-L1 cells were incubated with ryanodine (100 nM, MedChemExpress, Monmouth Junction, USA) or U73122 (1 *μ*M, MedChemExpress, Monmouth Junction, USA) for 1 h to block the endoplasmic reticulum Ca^2+^ channel. Then, the cells were washed 3 times with Hank's Balanced Salt Solution and incubated with 10 *μ*M fluo-4-AM at 37°C for 1 h. After incubation, cells were then rewashed 3 times. Nikon Eclipse Ti-s microscopy (Nikon, Tokyo, Japan) was used to observe fluorescence. Fluorescent data were acquired after excitation at 490 nm and intensity at 525 nm.

### 2.9. RNA Extraction and PCR Analysis

Methods used for the RNA extraction and PCR analysis have been described previously [17]. The relative expression of mRNAs was normalized with *β*-actin levels using the 2^-*ΔΔ*Ct^ method. 2^-*ΔΔ*Ct^ is defined as the ratio of the relative mRNA level between the experimental group and the control group. Primers were designed using Primer Premier 5 based on sequences of mouse genes obtained from NCBI. All the primers used in this study are shown in Table 1.

### 2.10. Western Blot Analysis

The method used for the western blot analysis has been described previously [18]. Band intensities were quantified by the Image J software. The antibodies and their dilutions used in this study are listed in Table 2.

### 2.11. Drug Affinity Responsive Target Stability (DARTS) Assay

DARTS experiments for identifying the targets of DHM were performed as previously reported [19]. In brief, 3T3-L1 cells were lysed and treated with DHM (10 nM or 1 *μ*M) for 1 hour at room temperature. Then, the mixture was digested with 0.01% protease for 30 min at room temperature. The digestion was stopped by directly add 5×SDS-PAGE loading buffer and inactivation by boiling 5 min. Protein samples were separated with 8%-15% SDS-polyacrylamide gels and analyzed by Coomassie blue staining and western blotting.

### 2.12. Statistical Analysis

**Figure 1:**
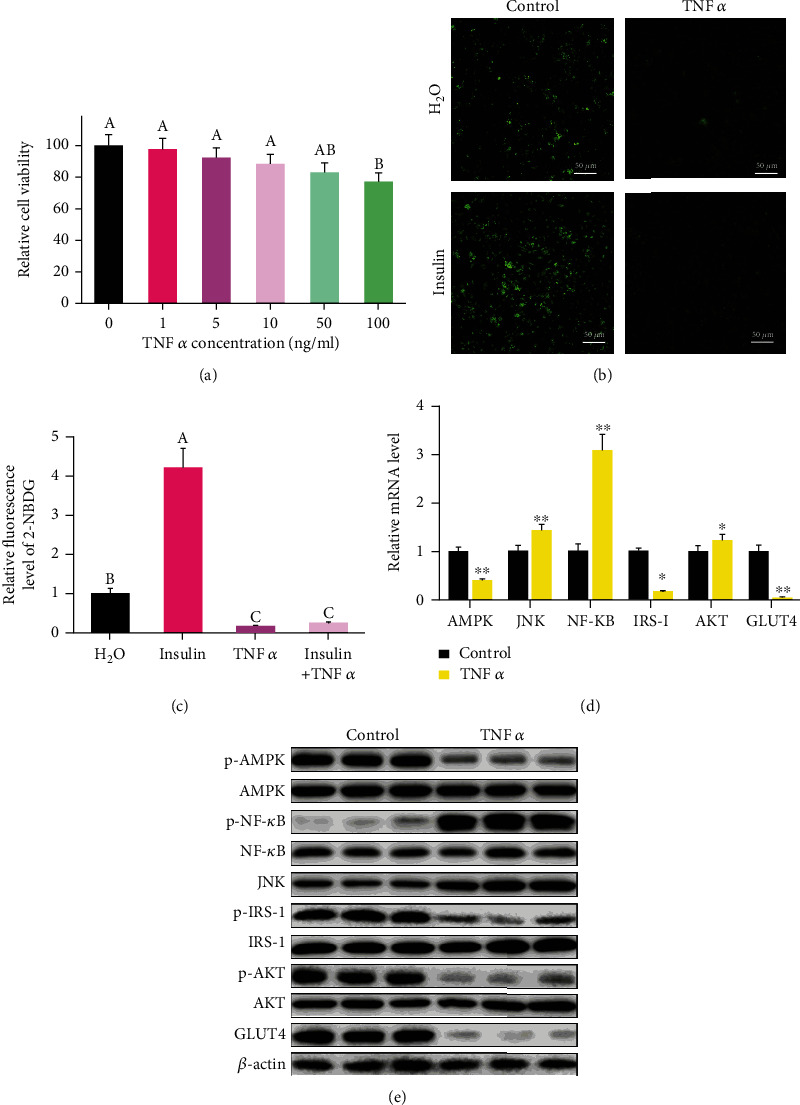
TNF*α* induced inflammatory response and insulin resistance in 3T3-L1 cells. (a) The optimal TNF*α* concentration used in 3T3-L1 experiments was determined by MTT. (b) The glucose uptake test confirmed that TNF*α* induced insulin resistance in 3T3-L1 cells. (c) Quantitative analysis of fluorescence intensity in (b) using the Image J software. (d) mRNA level of inflammation-induced insulin resistance-related genes was determined by qPCR. (e) The protein level of inflammation-induced insulin resistance-related genes was measured by WB. “p-” before the gene name means phosphorylated form. *N* = 6, ^∗^*p* < 0.05, and ^∗∗^*p* < 0.01. Any two bars with different letters indicate that these two groups are statistically significantly different (*p* < 0.05). Magnification was 100x. The scale bar on the photomicrographs represents 50 *μ*m.

**Figure 2:**
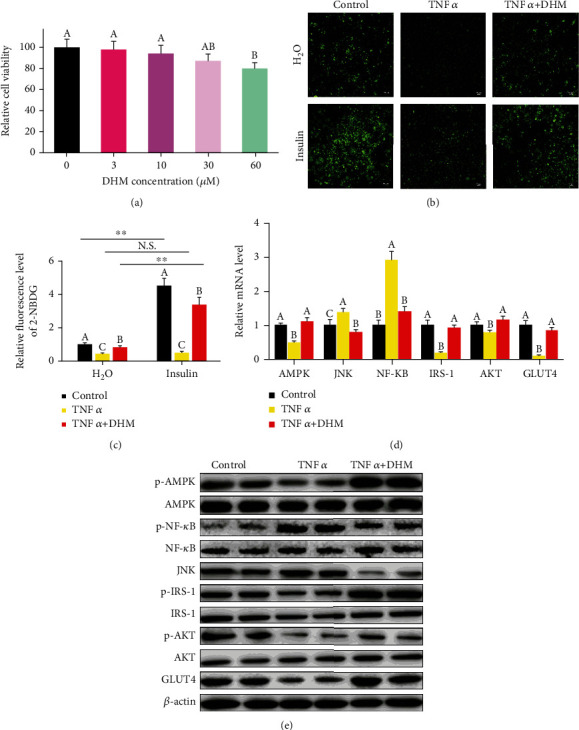
DHM (1 *μΜ*) ameliorated the inflammatory-induced insulin resistance. (a) The optimal DHM concentration used in 3T3-L1 was determined by MTT. (b) The glucose uptake test confirmed that TNF*α* induced insulin resistance in 3T3-L1 cells. (c) Quantitative analysis of fluorescence intensity in (b) using the Image J software. (d) The effect of DHM on the mRNA level of the inflammatory-induced insulin resistance-related genes was measured by qPCR. (e) The effect of DHM on the protein level of the inflammatory-induced insulin resistance-related genes was detected by WB. “p-” before the gene name means phosphorylated form. *N* = 6. ^∗∗^*p* < 0.01. Any two bars with different letters indicate that these two groups are statistically significantly different (*p* < 0.05). Magnification was 100x. The scale bar on the photomicrographs represents 50 *μ*m.

**Figure 3:**
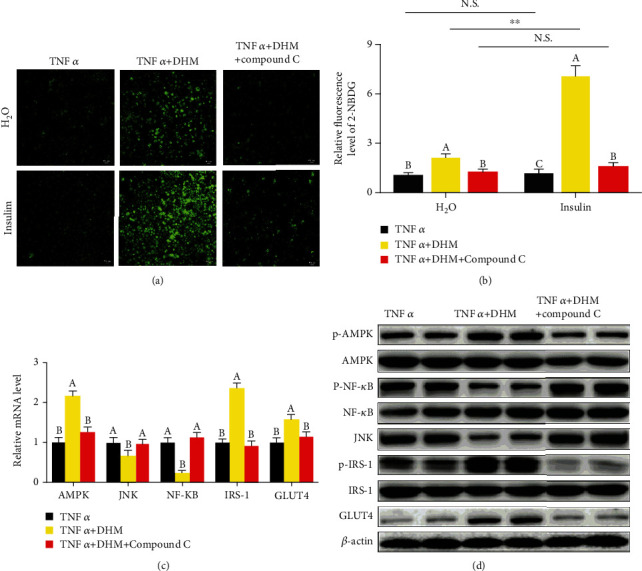
DHM (1 *μΜ*) ameliorated inflammation-induced insulin resistance through AMPK. (a) DHM alleviated TNF*α*-induced insulin resistance was dependent on AMPK, as shown by the glucose uptake test. (b) Quantitative analysis of fluorescence intensity in (a) using the Image J software. (c) DHM regulated the mRNA level of inflammatory response-induced insulin resistance-related genes was dependent on AMPK, as shown by qPCR. (d) DHM regulated the protein level of inflammatory response-induced insulin resistance-related genes was dependent on AMPK, as shown by WB. *N* = 6. ^∗∗^*p* < 0.01. Any two bars with different letters indicate that these two groups are statistically significantly different (*p* < 0.05). Magnification was 100x. The scale bar on the photomicrographs represents 50 *μ*m.

**Figure 4:**
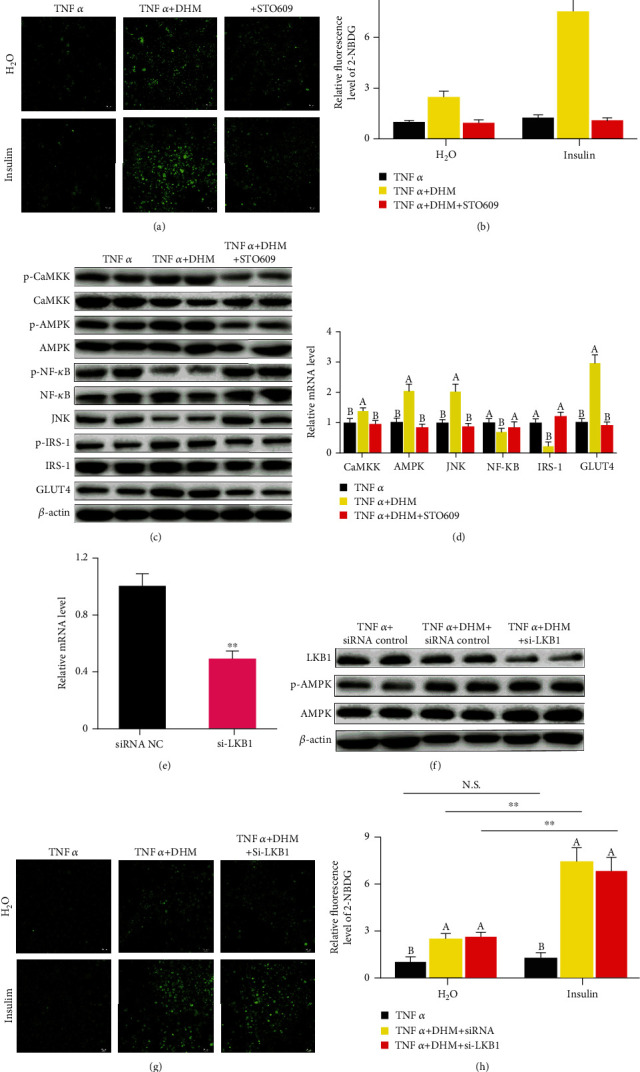
DHM (1 *μΜ*) ameliorated inflammation-induced insulin resistance through CaMKK-AMPK instead of the LKB1-AMPK pathway. (a) DHM alleviated TNF*α*-induced insulin resistance was dependent on CaMKK, as shown by the glucose uptake test. (b) Quantitative analysis of fluorescence intensity in (a) using the Image J software. (c) DHM regulated the protein level of inflammatory response-induced insulin resistance-related genes was dependent on CaMKK, as shown by WB. “p-” before the gene name means phosphorylated form. (d) DHM regulated the mRNA level of inflammatory response-induced insulin resistance-related genes was dependent on CaMKK, as shown by qPCR. (e) LKB1 was interfered with by small RNA, as shown by qPCR. (f) LKB1 interference did not affect the AMPK expression, as shown by WB. “p-” before the gene name means phosphorylated form. (g) LKB1 did not affect DHM improvement on TNF*α*-induced insulin resistance, as shown by the glucose uptake test. (h) Quantitative analysis of fluorescence intensity in (g) using the Image J software. *N* = 6, and ^∗∗^*p* < 0.01. Any two bars with different letters indicate that these two groups are statistically significantly different (*p* < 0.05). Magnification was 100x. The scale bar on the photomicrographs represents 50 *μ*m.

**Figure 5:**
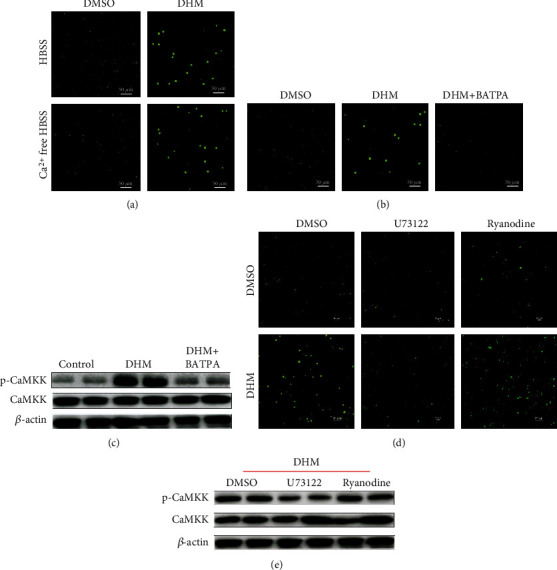
DHM (1 *μΜ*) activated CaMKK by increasing intracellular Ca^2+^ concentration through the PLC-IP3 receptor pathway. (a) DHM increased intracellular Ca^2+^ signal, as shown by the Ca^2+^ probe test. (b) After cellular Ca^2+^ was cleared, the activation effect of DHM on intracellular Ca^2+^ was eliminated, as shown by the Ca^2+^ probe test. (c) The effect of DHM on CaMKK activation depended on intracellular Ca^2+^ signal, as shown by WB. (d) DHM increased intracellular Ca^2+^ concentration was dependent on the PLC-IP3 receptor pathway, as shown by the Ca^2+^ probe test. (e) The effect of DHM on CaMKK activation depended on the intracellular PLC-IP3 receptor pathway, as shown by WB. *N* = 6.

All data are expressed as the mean ± standard error of the mean (SEM) of three independent experiments. Our data is a normal distribution, and the homogeneity of data between each treatment group is equal under the SPSS analysis. In Figures 1(d), 2(c), 3(b), 4(e) and 4(h), and 5(f) , the unpaired Student's *t*-test was used for *p* value calculations, where ∗ represents *p* < 0.05 and ∗∗ represents *p* < 0.01. Significant differences among groups (≥3) were determined by one-way ANOVA (SPSS v18.0, IBM Knowledge Center, Chicago, IL, USA). Multiple comparisons between the groups were performed using the S-N-K method. Any two bars with different letters indicate that these two groups are statistically significantly different (*p* < 0.05).

## 3. Results

### 3.1. DHM Reduced Fat Accumulation and Inflammation in HFD-Induced Obese Mice

**Figure 6:**
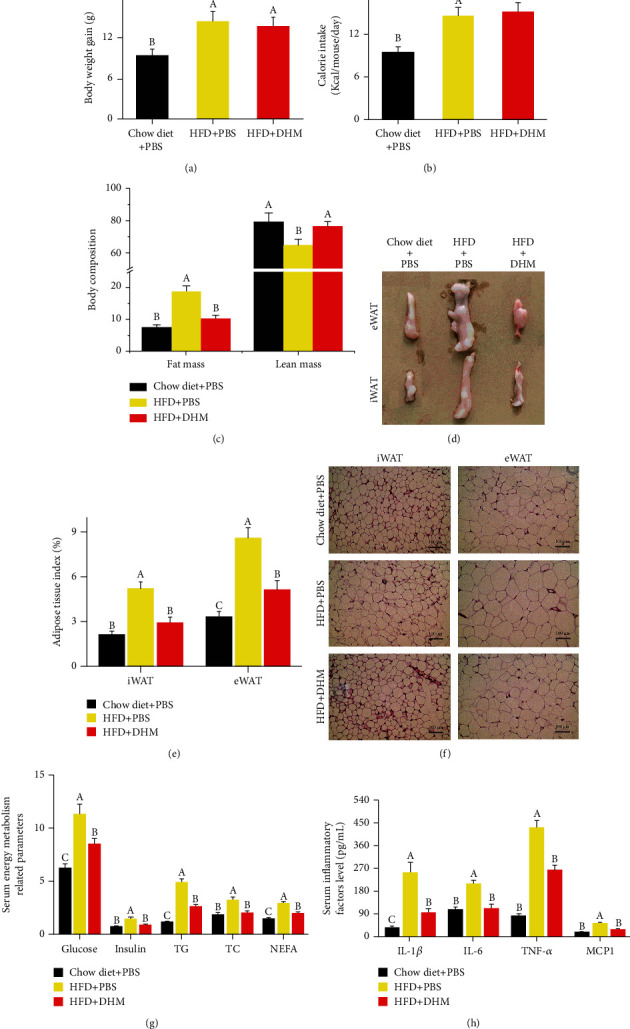
DHM reduced fat accumulation and inflammation in HFD-induced obese mice. (a) DHM did not affect HFD-induced body weight gain in mice. (b) DHM did not affect HFD-induced average daily energy intake. (c) DHM prevented HFD-induced fat gain and muscle loss. (d) Representative morphology image of mouse adipose tissue. (e) DHM inhibited HFD-induced adipose tissue hypertrophy, as shown by the percentage of adipose tissue index. (f) HFD induced adipocyte hypertrophy, and DHM prevented HFD-induced adipocyte hypertrophy, as shown by H&E staining. (g) DHM alleviated HFD-induced high levels of glucose, insulin, total triglycerides, total cholesterol, and free fatty acids in the serum. (h) DHM reduced the increase of the HFD-induced inflammatory cytokines in serum. *N* = 6. Any two bars with different letters indicate that these two groups are statistically significantly different (*p* < 0.05). Magnification was 200x. The scale bar on the photomicrographs represents 100 *μ*m.

Compared with the HFD group, HFD not only increased energy intake and body weight gain in mice (Figures 6(a) and 6(b)), but HFD also increased fat mass (Figures 6(c)), indicating the HFD-induced obese mouse model was constructed successfully. Compared with the HFD group, DHM did not affect energy intake and body weight gain (Figures 6(a) and 6(b)), but DHM prevented the increase of fat mass caused by HFD (Figure 6(c)). Both adipose tissue weight and adipose tissue index (percentages of iWAT weight/body weight or eWAT weight/body weight) were increased in HFD-induced obese mice compared with the control group (Figures 6(d) and 6(e) and Figure S1B). DHM reversed the HFD-induced eWAT and iWAT hypertrophy (Figures 6(d) and 6(e) and Figure S1B). We found HFD increased the area of adipocytes in iWAT and eWAT, while DHM inhibited the hypertrophy of adipocytes caused by HFD, based on the H&E results (Figure 6(f) and Figure S1B). For the energy metabolism-related parameters, HFD increased the levels of glucose, insulin, total triglycerides, total cholesterol, and free fatty acids in serum. DHM alleviated the increase of energy metabolism-related parameters in serum caused by HFD (Figure 6(g)). Since obesity is always accompanied by low-level inflammation, we also measured the level of the inflammatory cytokines in the serum. HFD induced a higher level of IL-1*β*, IL-6, TNF*α*, and MCP1 in obese mice, as measured by ELISA. DHM reduced the increase in the HFD-induced inflammatory cytokines in the serum (Figure 6(h)). These results indicate that DHM reduced fat accumulation and inflammation in HFD-induced obese mice.

### 3.2. DHM Ameliorated Insulin Resistance Induced by Inflammation in HFD Mice

**Figure 7:**
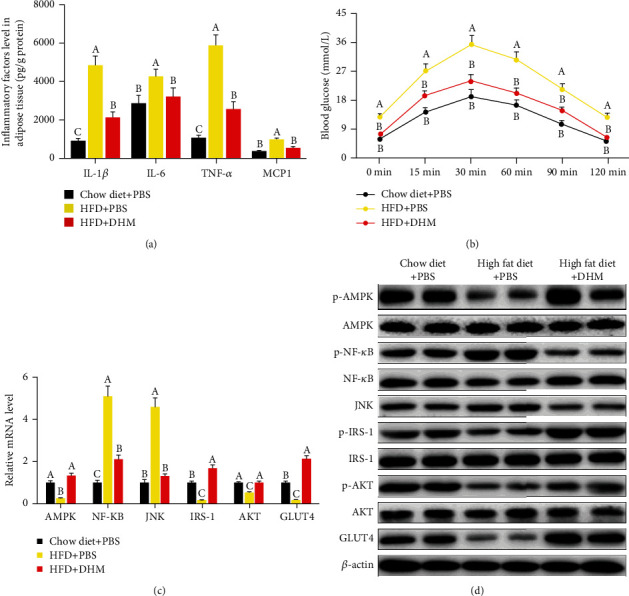
DHM ameliorated inflammation-induced insulin resistance in HFD mice. (a) HFD elevated the level of adipose tissue inflammatory cytokines in mice, and DHM suppressed the effect of HFD. (b) Oral glucose tolerance test results showed that DHM improved HFD-induced insulin resistance. (c) HFD inhibited the mRNA level of AMPK, IRS-1, AKT, and GLUT4. HFD increased the mRNA level of NF-*κ*B and JNK. DHM reversed the mRNA level of inflammation-induced insulin resistance-related genes. (d) HFD inhibited the protein level of p-AMPK, p-IRS-1, p-AKT, and GLUT4. HFD increased the protein level of p-NF-*κ*B and JNK. DHM reversed the protein level of inflammation-induced insulin resistance-related genes. “p-” before the gene name means phosphorylated form. *N* = 6. Any two bars with different letters indicate that these two groups are statistically significantly different (*p* < 0.05).

To investigate whether DHM ameliorated insulin resistance induced by inflammation, we measured the level of inflammatory cytokines in adipose tissue and insulin sensitivity in mice. ELISA results showed that HFD caused an increase in inflammatory cytokines IL-1*β*, IL-6, TNF*α*, and MCP1 in adipose tissue, while DHM reduced the level of adipose tissue inflammatory cytokines induced by HFD (Figure 7(a)). Through the oral glucose tolerance test, we found that the glucose clearance ability was decreased in the HFD-induced obese mice, while DHM enhanced the ability of glucose clearance in the HFD-fed mice (Figure 7(b)). qPCR and western blot results also showed that AMPK, an adipose tissue energy metabolism sensor, was inhibited in HFD-induced obese mice (Figures 7(c) and 7(d) and Figure S2). HFD-induced obesity activated the expression of NF-*κ*B and JNK. NF-*κ*B and JNK, they, in turn, inhibited the activation of IRS-1 and AKT, indicating that HFD induced insulin resistance (Figures 7(c) and 7(d) and Figure S2). The insulin resistance, subsequently, inhibited the expression of GLUT4, a transporter for glucose uptake (Figures 7(c) and 7(d) and Figure S2). However, DHM activated AMPK and inhibited NF-*κ*B and JNK induced by HFD. DHM reversed the inhibition on IRS-1 and AKT induced by HFD and finally increased GLUT4 expression (Figures 7(c) and 7(d) and Figure S2). These results indicate that DHM ameliorated inflammatory-induced insulin resistance in HFD mice.

### 3.3. TNF*α* Induced Inflammatory Response and Insulin Resistance in 3T3-L1 Cells

We constructed a cellular model of TNF*α*-induced insulin resistance in 3T3-L1 cells to establish the mechanism of DHM in relieving HFD-induced inflammation and insulin resistance. TNF*α*, less than 50 ng/mL, did not damage cell viability based on the result of MTT assay (Figure 1(a)). To simulate the low-level inflammatory response during obesity, we selected the TNF*α* concentration of 1 ng/mL for five days in subsequent experiments. After five days of TNF*α* treatment, insulin stimulation did not increase glucose uptake in 3T3-L1 cells, and the cells developed an insulin resistance phenotype (Figures 1(b) and 1(c)). qPCR and western blot results also showed that AMPK was inhibited after TNF*α* treatment (Figures 1(d) and 1(e) and Figure S3). TNF*α* treatment activated the expression of NF-*κ*B and JNK (Figures 1(d) and 1(e) and Figure S3). NF-*κ*B and JNK, they, in turn, inhibited IRS-1 and AKT, indicating that TNF*α* induced insulin resistance (Figures 1(d) and 1(e) and Figure S3). The insulin resistance, subsequently, inhibited the expression of GLUT4 (Figures 1(d) and 1(e) and Figure S3). Thus, these results suggest that TNF*α* activated the inflammatory response and induced insulin resistance.

### 3.4. DHM Ameliorated the Inflammatory-Induced Insulin Resistance

We then investigated whether DHM prevented insulin resistance induced by the inflammatory response. DHM, less than 30 *μ*M, did not damage cell viability based on MTT assay (Figure 2(a)). Insulin stimulation did not increase glucose uptake in 3T3-L1 cells after TNF*α* treatment (Figures 2(b) and 2(c)), while DHM prevented the TNF*α*-induced insulin resistance (Figures 2(b) and 2(c)). Compared with TNF*α* treatment, cotreatment with TNF*α* and DHM, AMPK was activated, and the expression of NF-*κ*B and JNK was inhibited, as shown by the qPCR and WB results (Figures 2(d) and 2(e) and Figure S4). Compared with TNF*α* treatment, DHM activated IRS-1 indicating that DHM ameliorated the TNF*α*-induced insulin resistance (Figures 2(d) and 2(e) and Figure S4). The improvement of insulin sensitivity, subsequently, increased the expression of GLUT4 (Figures 2(d) and 2(e)and Figure S4). Thus, the above results indicate that DHM ameliorated insulin resistance induced by inflammation.

### 3.5. DHM Ameliorated Inflammation-Induced Insulin Resistance through AMPK

We blocked AMPK activity using Compound C to investigate whether DHM exerted its anti-insulin resistance function through AMPK in 3T3-L1 cells. The ability of DHM to improve TNF*α*-induced insulin resistance was blocked by inhibiting AMPK, as shown by the glucose uptake assay (Figures 3(a) and 3(b)). When Compound C blocked AMPK activity, the activation effect of DHM on AMPK and IRS-1 disappeared (Figures 3(c) and 3(d) and Figure S5), and the promotion effect of DHM on the expression of GLUT4 also disappeared (Figures 3(c) and 3(d) and Figure S5). Blocked AMPK, the DHM inhibitory effect on NF-*κ*B and JNK was eliminated (Figures 3(c) and 3(d) and Figure S5). These results indicate that DHM ameliorated inflammation-induced insulin resistance in 3T3-L1 cells through AMPK.

### 3.6. DHM Ameliorated Inflammation-Induced Insulin Resistance through CaMKK-AMPK instead of LKB1-AMPK Pathway

We blocked the AMPK upstream factors CaMKK and LKB1 to investigate the pathway DHM used to activate AMPK. When STO-609 blocked CaMKK activity, the ability of DHM to improve TNF*α*-induced insulin resistance disappeared, as shown by the glucose uptake assay (Figures 4(a) and 4(b)). When STO-609 blocked CaMKK activity, the activation effect of DHM on CaMKK, AMPK, and IRS disappeared (Figures 4(c) and 4(d) and Figure S6B) and the promotion effect of DHM on the expression of GLUT4 also disappeared (Figures 4(c) and 4(d) and Figure S6B). Blocked CaMKK also caused DHM to lose its inhibitory effect on NF-*κ*B and JNK (Figures 4(c) and 4(d) and Figure S6B). We then interfered with LKB1 expression using siRNA (Figures 4(e) and 4(f)). LKB1 interference did not affect the activation of AMPK by DHM (Figure 4(f) and Figure S6A). LKB1 interference did not affect the improvement of DHM on TNF*α*-induced insulin resistance, as shown by the glucose uptake assay (Figures 4(g) and 4(h)). These results indicate that DHM ameliorated inflammation-induced insulin resistance in 3T3-L1 cells through CaMKK-AMPK instead of the LKB1-AMPK pathway.

### 3.7. DHM Activated CaMKK by Increasing Intracellular Ca^2+^ Concentration through PLC-IP3 Receptor Pathway

CaMKK is regulated by cellular Ca^2+^, so we investigated whether DHM activated cellular Ca^2+^ signal. DHM treatment activated intracellular Ca^2+^ signal, as shown by the Ca^2+^ probe test. When Ca^2+^ was removed from the extracellular medium, DHM still increased intracellular Ca^2+^ level (Figure 5(a)). After the cellular Ca^2+^ was cleared by the intracellular Ca^2+^ chelating agent BATAP-AM, the activation effect of DHM on intracellular Ca^2+^ was eliminated, indicating that DHM activation on intracellular Ca^2+^ was dependent on intracellular Ca^2+^ storage (Figure 5(b)). After cellular Ca^2+^ was cleared, DHM could no longer activate CaMKK, as shown by the WB results (Figure 5(c) and Figure S7A). Endoplasmic reticulum was the major organelle for Ca^2+^ release and recovery in cells. We used U73122, a phospholipase C (PLC) inhibitor, to block the endoplasmic reticulum Ca^2+^ channel IP3 receptor by preventing phospholipid phosphatidylinositol 4,5-diphosphate (PIP2) from degrading into inositol 1,4,5-triphosphate (IP3). We also used ryanodine to block the Ca^2+^ channel ryanodine receptor. Blocking the ryanodine receptor did not affect cellular activation of intracellular Ca^2+^ signal induced by DHM (Figure 5(d)). After the PLC-IP3 receptor pathway was blocked, the DHM function on activating the intracellular Ca^2+^ signal disappeared (Figure 5(d)). After blocking the PLC-IP3 receptor pathway, DHM failed to activate CaMKK, as shown by the WB results (Figure 5(e) and Figure S7B). These results indicate that DHM activated CaMKK by increasing intracellular Ca^2+^ concentration through the PLC-IP3 receptor pathway.

### 3.8. DHM Ameliorated Inflammatory-Induced Insulin Resistance through Interaction with PLC

**Figure 8:**
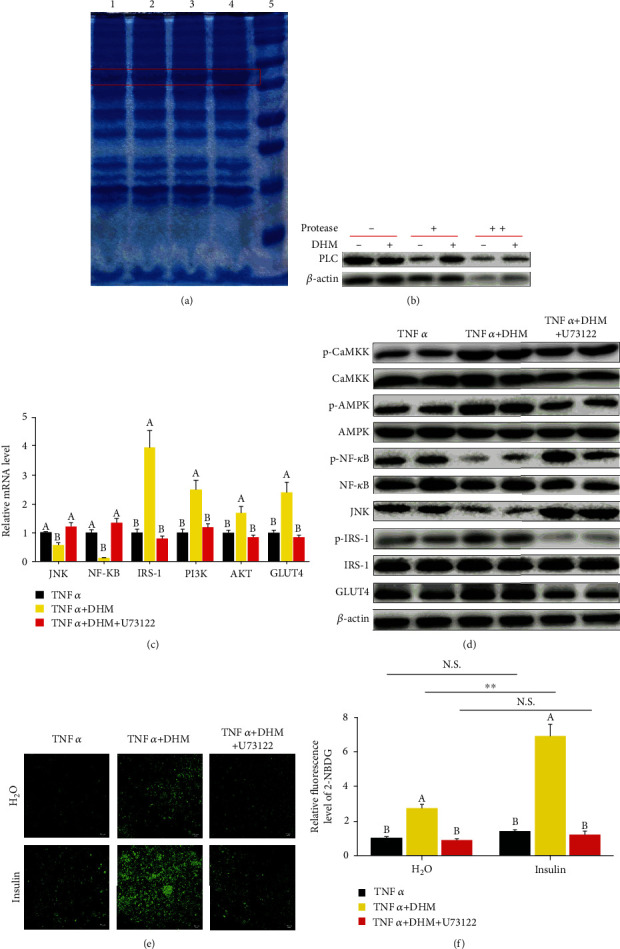
DHM (1 *μΜ*) ameliorated inflammatory-induced insulin resistance through interaction with PLC. (a) DHM might interact with the PLC, as shown by DARTS, and lines 1 to 5 represent molecular weight marker, DMSO, 10 nM DHM, and 1 *μ*M DHM, respectively. (b) DHM promoted the stability of PLC protein, as shown by WB. (c) The effect of DHM on the mRNA level of inflammatory response-induced insulin resistance-related genes was dependent on the PLC-IP3 receptor pathway, as shown by qPCR. (d) The effect of DHM on the protein level of inflammatory response-induced insulin resistance-related genes was dependent on the PLC-IP3 receptor pathway, as shown by WB. “p-” before the gene name means phosphorylated form. (e) DHM alleviated TNF*α*-induced insulin resistance was dependent on the PLC-IP3 receptor pathway, as shown by the glucose uptake test. (f) Quantitative analysis of fluorescence intensity in (e) using the Image J software. *N* = 6. ^∗∗^*p* < 0.01. Any two bars with different letters indicate that these two groups are statistically significantly different (*p* < 0.05). Magnification was 100x. The scale bar on the photomicrographs represents 50 *μ*m.

We verified the interaction of DHM and PLC by drug affinity responsive target stability (DARTS) assay to investigate the pathway DHM used to activate the PLC-IP3 receptor pathway. From the DARTS results, we found a brighter band at the position of about 50 KD SDS-PAGE gel in the DHM treatment group, suggesting that DHM might interact with the PLC (Figure 8(a)). At the same time, we also detected the DARTS product through WB. DHM promoted the stability of PLC protein, as shown by the WB results, indicating that DHM interacted with PLC protein (Figure 8(b) and Figure S8A). When U73122 blocked the PLC-IP3 receptor pathway, the activation effect of DHM on CaMKK, AMPK, and IRS-1 disappeared (Figure 8(c)), and the promotion effect of DHM on the expression of GLUT4 also disappeared (Figures 8(c) and 8(d) and Figure S8B). Blocked PLC-IP3 receptor pathway also caused DHM to lose its inhibitory effect on JNK and NF-*κ*B (Figures 8(c) and 8(d) and Figure S8B). When U73122 blocked the PLC-IP3 receptor pathway, the ability of DHM to improve TNF*α*-induced insulin resistance disappeared, as shown by the glucose uptake assay (Figures 8(e) and 8(f)). These results indicate that DHM ameliorated inflammatory-induced insulin resistance through interaction with the PLC.

## 4. Discussion

Increasing incidence of metabolic syndrome-related diseases such as obesity, diabetes, and cardiovascular diseases has become a global health problem. Insulin resistance is the common pathophysiology basis of metabolic syndrome. All metabolic syndrome patients have insulin resistance in different degrees. Therefore, therapeutic strategies for metabolic syndrome need to ameliorate insulin resistance. In this study, we found that DHM activated the Ca^2+^-CaMKK-AMPK signal pathway by binding to the PLC. Activation of AMPK ameliorated adipocytes insulin resistance by blocking the inflammation factor-induced inflammatory response. Our study not only clarified the molecular mechanism of DHM in inflammation-induced insulin resistance but also discovered the target of DHM in this process. This study offers a new therapeutic strategy for patients with metabolic syndrome.

The increasing incidence of insulin resistance is inextricably related to the increase in the global obese population [20]. A recent report showed that 2.1 billion people, about 30% of the world population, were either overweight or obese. During the process of obesity, the proliferation and hypertrophy of adipocytes triggered hypoxic stress, causing the secretion of inflammatory cytokines [21]. Our results showed that levels of serum and adipose tissue inflammatory cytokines were increased in HFD-induced obese mice.

The inflammatory cytokines secreted by adipose tissue activated NF-*κ*B and JNK signal pathways in insulin-sensitive organs like adipose tissue, skeletal muscle, and liver [22–24]. The activation of NF-*κ*B and JNK inhibited the phosphorylation of IRS-1 and, in turn, blocked the PI3K/AKT activation [25]. Results from our in vivo experiments showed that the level of the inflammatory cytokines was increased in adipose tissue; the inflammatory cytokines induced the NF-*κ*B and JNK-1 activation and insulin resistance in mice. Results from our in vitro experiments also showed that TNF*α* induced the NF-*κ*B and JNK-1 activation and insulin resistance in 3T3-L1 cells.

AMPK is not only an important regulator in cellular energy metabolism but also plays an essential role in many physiological processes such as tumor growth, inflammation, and enhanced insulin sensitivity [26]. The pharmacological function of DHM to activate AMPK has been demonstrated in various physiological processes. DHM improved skeletal muscle insulin sensitivity partially through inducing autophagy by activating the AMPK-PGC-1*α*-Sirt signaling pathway [14]. DHM protected rats from developing Alzheimer's disease via the upregulation of the AMPK-SIRT1 pathway to inhibit inflammation-induced apoptosis of hippocampal cells and ameliorate cognitive function [27]. We demonstrated that DHM activated AMPK *in vivo* and *in vitro*, and we also demonstrated that the function of DHM in ameliorating insulin resistance depended on its activation effect on AMPK *in vitro.*

AMPK blocked the inflammatory response by inhibiting the NF-*κ*B signaling pathway. AMPK activation blocked the phosphorylation of IKK and NF-*κ*B directly [28]. AMPK also activated SIRT1; then, P65, the subunit of NF-*κ*B, was deacetylated by SIRT1. Thus, the transcriptional activity of NF-*κ*B was also inhibited [29]. In this study, we found that DHM blocked NF-*κ*B phosphorylation to ameliorate insulin resistance through AMPK *in vivo* and *in vitro*.

To our knowledge, most DHM-related research reported that DHM functions depend on its activation of AMPK [30]; but AMPK is not the direct target of DHM in cells, and the target of DHM in cells has not been identified. Two predominant upstream kinases are known to activate AMPK: LKB1 and CaMKK [31]. Cynandione A, a Cynanchum wilfordii extract, inhibited lipogenesis by activating the LKB1-AMPK pathway in HepG2 cells [32]. Apigenin, a natural flavonoid, induced autophagy in human keratinocytes via upregulating CaMKK-AMPK [33]. To investigate the pathway DHM used to activate AMPK, we blocked the AMPK upstream factors LKB1 and CaMKK. Results from our *in vitro* experiment showed that DHM ameliorated inflammation-induced insulin resistance through CaMKK-AMPK instead of the LKB1-AMPK pathway.

As far as we know, there is no published report on how DHM activates CaMKK. Triptolide, an extract from the Chinese herb thunder god vine, increased intracellular Ca^2+^ by stimulating the endoplasmic reticulum stress. The triptolide induced autophagy in human prostate cancer cells by activating the Ca^2+^-CaMKK-AMPK signaling pathway [34]. Brosimone I, a flavonoid from Artocarpus heterophyllus, increased intracellular Ca^2+^ by stimulating the endoplasmic reticulum stress. Brosimone I induced cell cycle arrest and apoptosis via the activation of the Ca^2+^-CaMKK-AMPK signaling pathway [35]. These reports showed that plant extracts could activate the CaMKK-AMPK signaling pathway through Ca^2+^. In this study, we found that the DHM activated CaMKK-AMPK signaling pathway is dependent on Ca^2+^. Dandelion root extract (10-400 *μ*g/mL) dose-dependently increased intracellular Ca^2+^ level in the presence of external Ca^2+^. The effect of dandelion root extract-induced Ca^2+^ increase was inhibited in the absence of extracellular Ca^2+^ [36]. However, in our study, DHM activated intracellular Ca^2+^ signals regardless of the presence or absence of extracellular Ca^2+^. The endoplasmic reticulum is the main storage organelle for Ca^2+^ in cells. The ryanodine receptor and IP3 receptor are the main channels for calcium release from the endoplasmic reticulum and play a central role in cellular Ca^2+^ signal transduction [37]. In this study, we discovered that by blocking the PLC-IP3 receptor pathway, the DHM's ability to activate Ca^2+^ and CaMKK disappeared, but blocking the ryanodine receptor had no effect on preventing the activation of Ca^2+^-CaMKK induced by DHM.

The identification of target protein for small molecules is critical for drug discovery and chemical metabolomics [38]. DARTS is a quick and straightforward approach to identify potential target protein of small molecules. The mechanism of DARTS is that the interaction of small molecule and target protein resists proteolysis. The most significant advantage of this method is it allows the use of the small native molecule without any modification, such as the incorporation of biotin, photoaffinity labels, or radioisotope [39]. In this study, we used DARTS to identify potential binding targets of DHM and used DARTS-western blotting to test and validate the potential DHM target. Our results showed that DHM interacted with PLC to activate the Ca^2+^-CaMKK-AMPK signal pathway.

## 5. Conclusion

**Figure 9:**
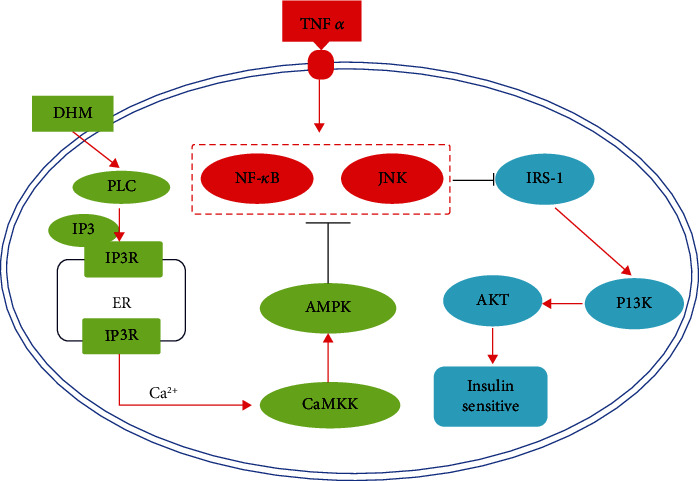
Summary model of DHM improves inflammation-induced insulin resistance via phospholipase C-CaMKK-AMPK signal pathway.

In conclusion, our results not only showed that DHM ameliorated inflammation-induced insulin resistance but also demonstrated that DHM activated the Ca^2+^-CaMKK-AMPK signal pathway through interacting with its target protein phospholipase C (Figure 9). Our results provided experimental data for the development of DHM as a functional food additive and a new therapeutic strategy for preventing insulin resistance.

## Figures and Tables

**Table 1:** The primers used in this study.

Gene name	Forward primer sequence (5′-3′)	Reversed primer sequence (5′-3′)
*β*-Actin	TCGTACCACTGGCATTGTGAT	CGAAGTCTAGGGCAACATAG
NF-*κ*B	ATGCCAGTGAGAATGTATGC	ACGCAGGAGACGGAAGAAT
IRS-1	ATGTCGCCAGTGGGAGATT	CTTCGGCAGTTGCGGTATA
PI3K	GACCAATACTTGATGTGGCTG	TCCCTCGCAATAGGTTCTCC
AKT	GCCCGCTTCTATGGTGC	CTTCTCGTGGTCCTGGTTGTA
JNK	CCAGCACCCATACATCAAC	TTCCTCCAAATCCATTACCTCC
GLUT4	AGTATGTTGCGGATGCTATGG	CTGCTCTAAAAGGTAAGGTGT

**Table 2:** The details of antibodies used in this study.

Primary antibody	Clone	Company	Catalog No.	Dilution
PLC	Polyclonal	Abcam	Ab41433	1 : 2000
CaMKK	Polyclonal	Abbkine	ABP53531	1 : 1000
p-CaMKK	Polyclonal	Affinit	AF4487	1 : 1000
LKB1	Polyclonal	Bioss	bs-3948R	1 : 2000
AMPK	Polyclonal	Abbkine	ABP50650	1 : 1000
p-AMPK	Polyclonal	Abbkine	ABP50452	1 : 1000
JNK	Polyclonal	ZEN BIO	380556	1 : 2000
NF-*κ*B	Polyclonal	Bioss	bsm-33117M	1 : 5000
p-NF-*κ*B	Polyclonal	Bioss	bs-3485R	1 : 500
IRS-1	Polyclonal	Abcam	341420	1 : 1000
p-IRS-1	Polyclonal	Abcam	Ab3690	1 : 2000
AKT	Monoclonal	CST	# 9272S	1 : 2000
p-AKT	Monoclonal	CST	# 4058S	1 : 2000
PI3K	Polyclonal	ZEN BIO	385369	1 : 1000
p-PI3K	Polyclonal	ZEN BIO	530854	1 : 1000
GLUT4	Polyclonal	Abcam	ab109313	1 : 2000
*β*-Actin	Monoclonal	Bioworld	BS6007M	1 : 5000
Secondary antibody	Conjugate used	Company	Catalog No.	Dilution
Goat anti-rabbit IgG	HRP	Bioss	bs-0295G	1 : 5000
Goat anti-mouse IgG	HRP	Bioworld	BS12478	1 : 50000

## Data Availability

The data and materials supporting the conclusions are included within the article.

## Abbreviations

AKT: Protein kinase B
DARTS: Drug affinity responsive target stability
DHM: Dihydromyricetin
eWAT: Epididymis white adipose tissue
H&E: Hematoxylin-eosin staining
HFD: High-fat diet
IP3: Inositol 1,4,5-triphosphate
IRS: Insulin receptor substrate
iWAT: Inguinal white adipose tissue
JNK: c-Jun NH2-terminal kinase
MTT: Methyl thiazolyl diphenyl-tetrazolium bromide
NF-*κ*B: Nuclear factor-kappa B
PI3K: Phosphatidylinositol 3-kinase
PIP2: Phosphatidylinositol 4,5-diphosphate
PLC: Phospholipase C
TNF*α*: Tumor necrosis factor *α*.

## Supplementary Materials

Supplementary Materials Figure S1: DHM reversed the HFD-induced eWAT and iWAT hypertrophy. (A) The quantitative data of mouse adipose tissue weight. (B) The quantitative data of the adipocyte average area. *N* = 6. Any two bars with different letters indicate that these two groups are statistically significantly different (*p* < 0.05). Figure S2: grayscale scanning statistics of inflammatory-induced insulin resistance-related proteins in Figure 7(d). *N* = 6. Any two bars with different letters indicate that these two groups are statistically significantly different (*p* < 0.05). Figure S3: grayscale scanning statistics of inflammatory-induced insulin resistance-related proteins in Figure 1(e). *N* = 6. ^∗^*p* < 0.05 and ^∗∗^*p* < 0.01. Figure S4: grayscale scanning statistics of inflammatory-induced insulin resistance-related proteins in Figure 2(e). *N* = 6. Any two bars with different letters indicate that these two groups are statistically significantly different (*p* < 0.05). Figure S5: grayscale scanning statistics of inflammatory-induced insulin resistance-related proteins in Figure 3(d). *N* = 6. Any two bars with different letters indicate that these two groups are statistically significantly different (*p* < 0.05). Figure S6: statistical results related to Figure 4. (A) Grayscale scanning statistics of p-AMPK and LKB1 protein in Figure 4(f). (B) Grayscale scanning statistics of inflammatory-induced insulin resistance-related proteins in Figure 4(c). *N* = 6. Any two bars with different letters indicate that these two groups are statistically significantly different (*p* < 0.05). Figure S7: statistical results related to Figure 5. (A) Grayscale scanning statistics of p-CaMKK protein level in Figure 5(c). (B) Grayscale scanning statistics of p-CaMKK protein level in Figure 5(e). *N* = 6. Any two bars with different letters indicate that these two groups are statistically significantly different (*p* < 0.05). Figure S8: statistical results related to Figure 8. (A) Grayscale scanning statistics of PLC protein level in Figure 8(b). (B) Grayscale scanning statistics of inflammatory-induced insulin resistance-related protein level in Figure 8(d). *N* = 6. ^∗∗^*p* < 0.01. Any two bars with different letters indicate that these two groups are statistically significantly different (*p* < 0.05).
