# Supplementary material for: Dihydromyricetin Ameliorates Inflammation-Induced Insulin Resistance via Phospholipase C-CaMKK-AMPK Signal Pathway
Source: Oxid Med Cell Longev. 2021 Oct 5;2021:8542809. doi: 10.1155/2021/8542809 (PMC8510796; doi:10.1155/2021/8542809)
Supplement: Supplementary Materials — Figure S1: DHM reversed the HFD-induced eWAT and iWAT hypertrophy. (A) The quantitative data of mouse adipose tissue weight. (B) The quantitative data of the adipocyte average area. N = 6. Any two bars with different letters indicate that these two groups are statistically significantly different (p < 0.05). Figure S2: grayscale scanning statistics of inflammatory-induced insulin resistance-related proteins in Figure 7(d). N = 6. Any two bars with different letters indicate that these two groups are statistically significantly different (p < 0.05). Figure S3: grayscale scanning statistics of inflammatory-induced insulin resistance-related proteins in Figure 1(e). N = 6. ∗p < 0.05 and ∗∗p < 0.01. Figure S4: grayscale scanning statistics of inflammatory-induced insulin resistance-related proteins in Figure 2(e). N = 6. Any two bars with different letters indicate that these two groups are statistically significantly different (p < 0.05). Figure S5: grayscale scanning statistics of inflammatory-induced insulin resistance-related proteins in Figure 3(d). N = 6. Any two bars with different letters indicate that these two groups are statistically significantly different (p < 0.05). Figure S6: statistical results related to Figure 4. (A) Grayscale scanning statistics of p-AMPK and LKB1 protein in Figure 4(f). (B) Grayscale scanning statistics of inflammatory-induced insulin resistance-related proteins in Figure 4(c). N = 6. Any two bars with different letters indicate that these two groups are statistically significantly different (p < 0.05). Figure S7: statistical results related to Figure 5. (A) Grayscale scanning statistics of p-CaMKK protein level in Figure 5(c). (B) Grayscale scanning statistics of p-CaMKK protein level in Figure 5(e). N = 6. Any two bars with different letters indicate that these two groups are statistically significantly different (p < 0.05). Figure S8: statistical results related to Figure 8. (A) Grayscale scanning statistics of PLC protein level i [file 8542809.f1.doc]

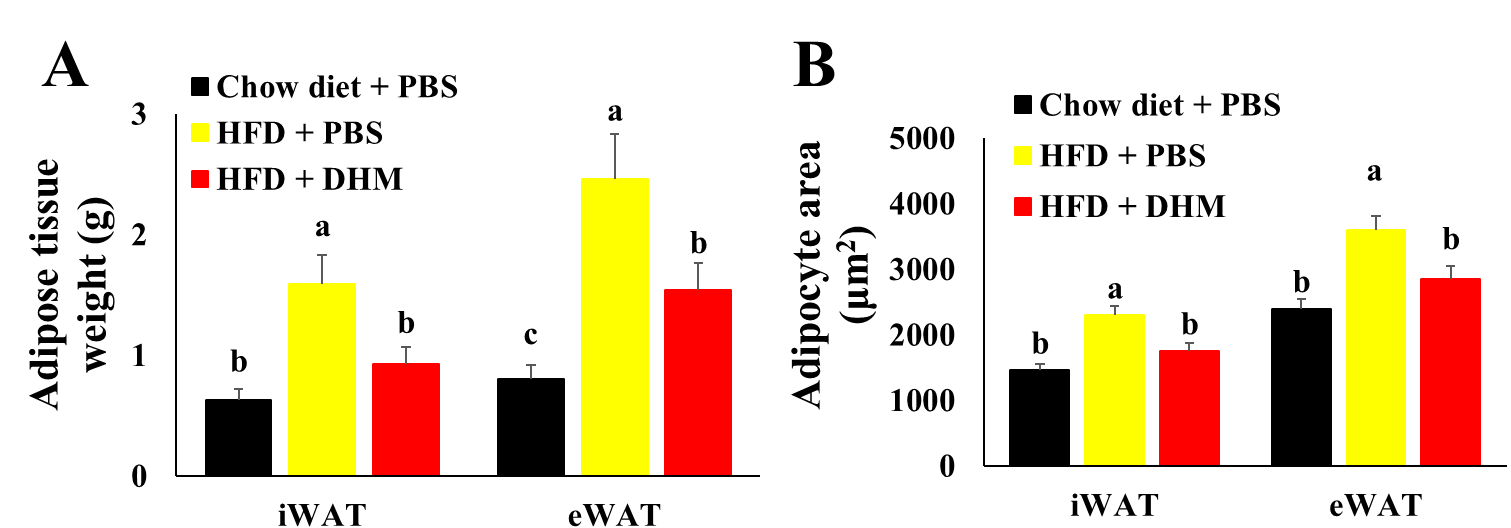


Figure S1. DHM reversed the HFD-induced eWAT and iWAT hypertrophy. A. The quantitative data of mice adipose tissue weight. B. The quantitative data of the adipocyte average area. N = 6. Any two bars with different letters indicate that these two groups are statistically significantly different (*p*<0.05).


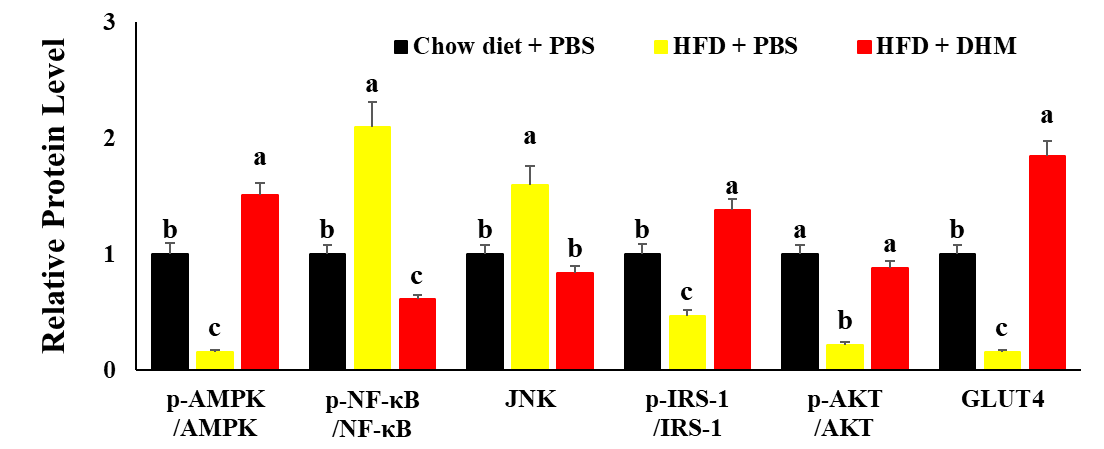


Figure S2. Grayscale scanning statistics of inflammatory induced insulin resistance-related proteins in Figure 2D. N = 6. Any two bars with different letters indicate that these two groups are statistically significantly different (*p*<0.05).


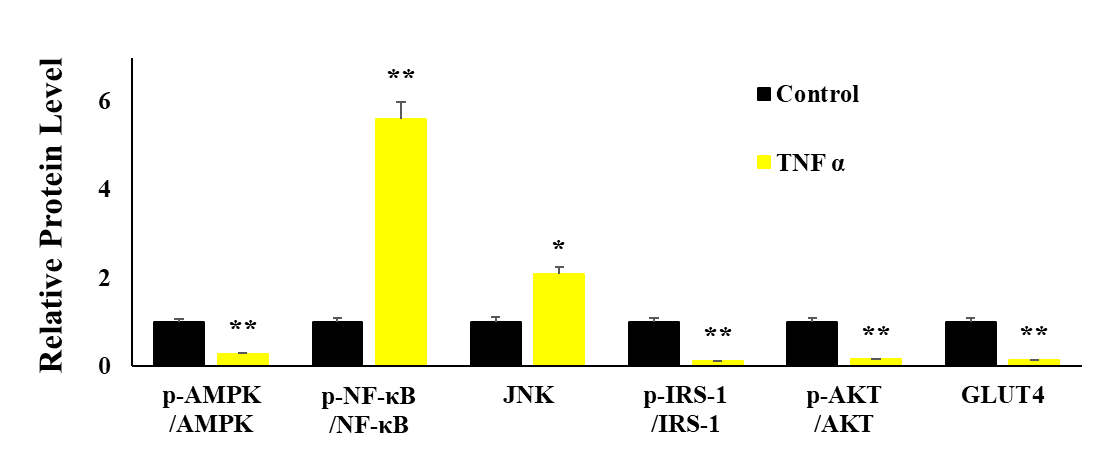


Figure S3. Grayscale scanning statistics of inflammatory induced insulin resistance-related proteins in Figure 3E. N = 6. * *p* < 0.05, ** *p* < 0.01.


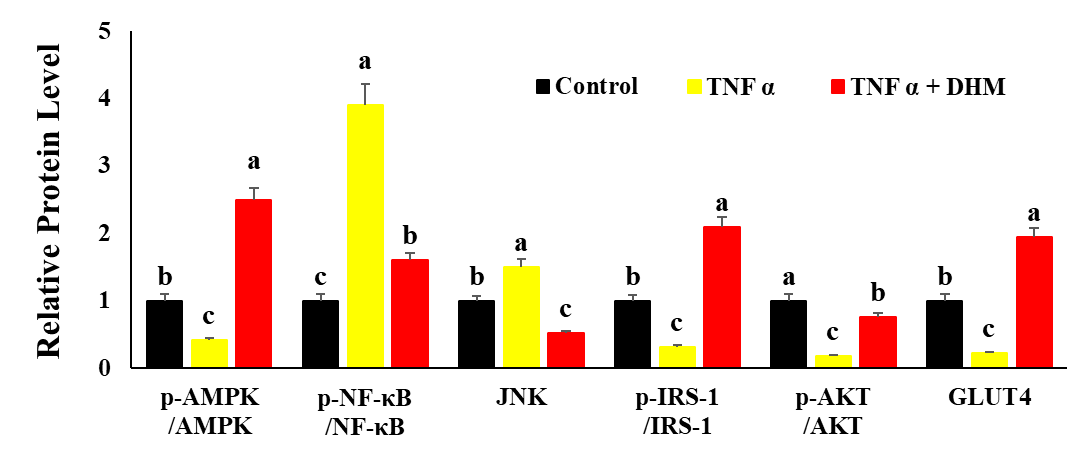


Figure S4. Grayscale scanning statistics of inflammatory induced insulin resistance-related proteins in Figure 4E.

N = 6. Any two bars with different letters indicate that these two groups are statistically significantly different (*p*<0.05).


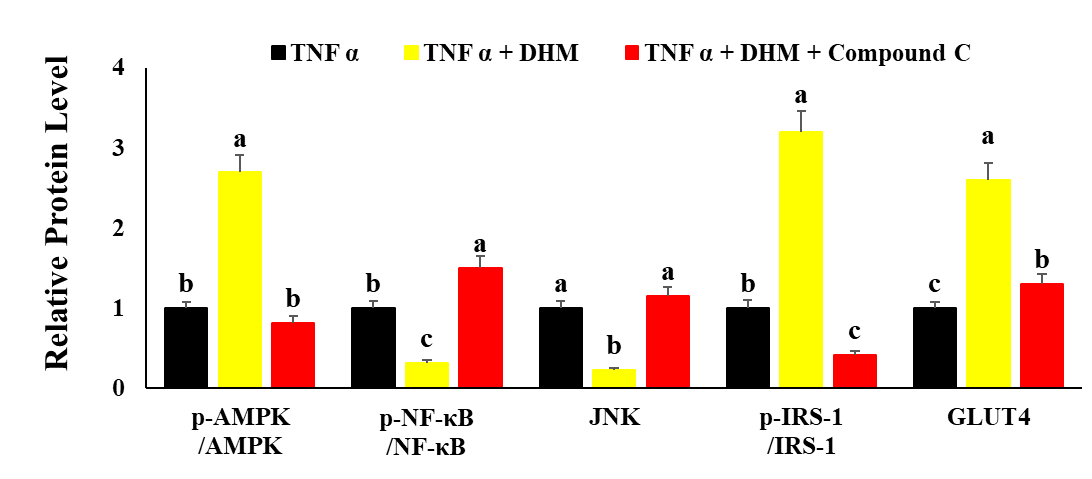


Figure S5. Grayscale scanning statistics of inflammatory induced insulin resistance-related proteins in Figure 5D.

N = 6. Any two bars with different letters indicate that these two groups are statistically significantly different (*p*<0.05).


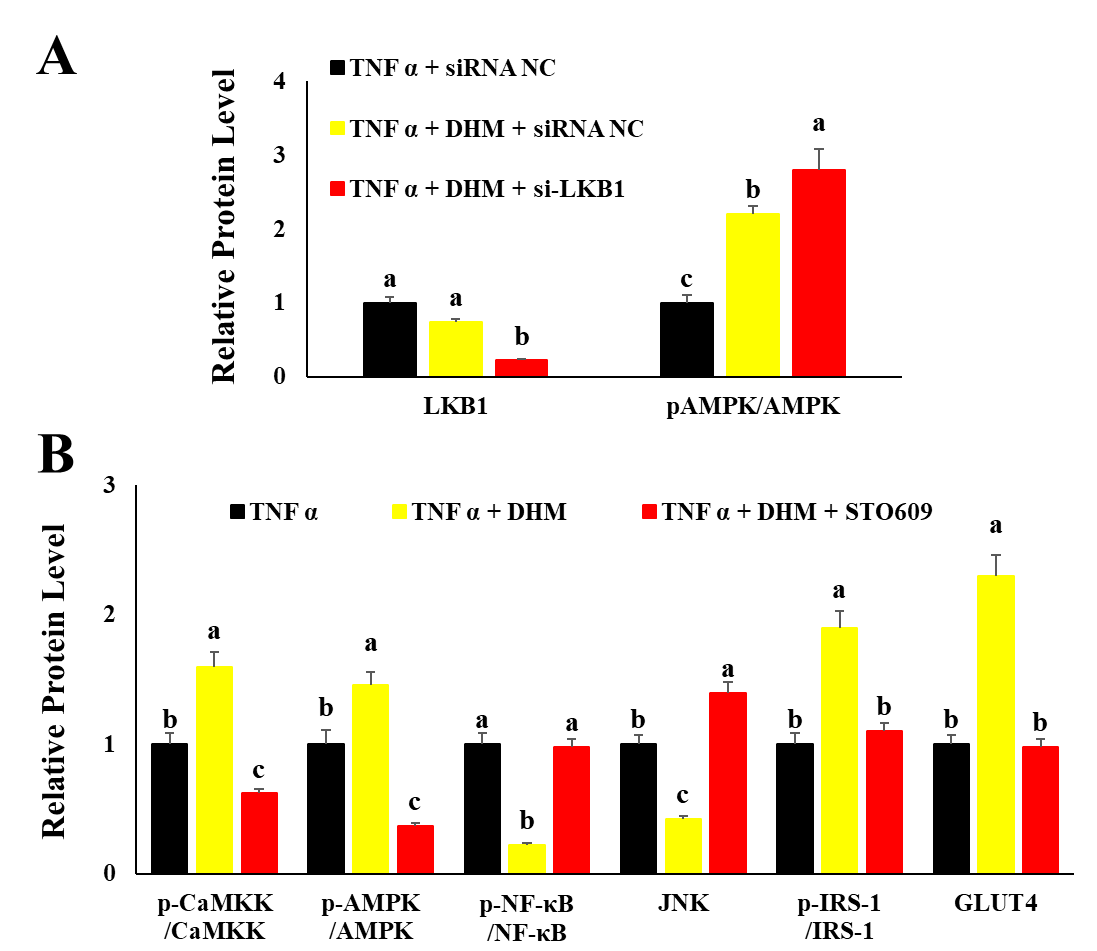


Figure S6. Statistical results related to Figure 6. A. Grayscale scanning statistics of p-AMPK and LKB1 protein in Figure 6F. B. Grayscale scanning statistics of inflammatory induced insulin resistance-related proteins in Figure 6C. N = 6. Any two bars with different letters indicate that these two groups are statistically significantly different (*p*<0.05).


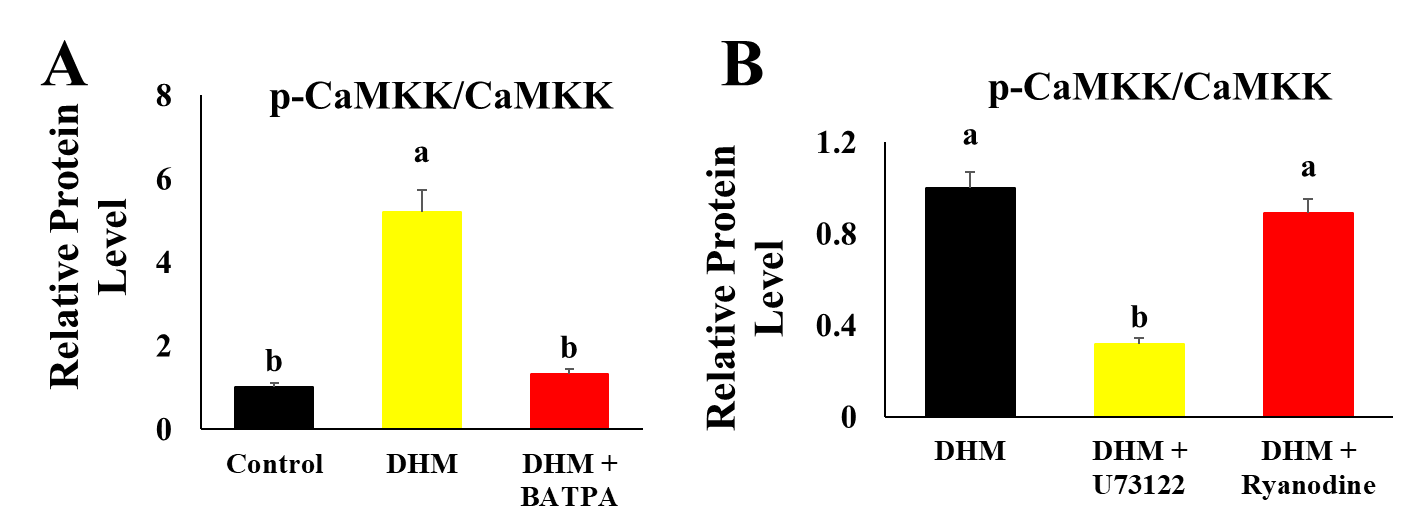


Figure S7. Statistical results related to Figure 7. A. Grayscale scanning statistics of p-CaMKK protein level in Figure 7C. B. Grayscale scanning statistics of p-CaMKK protein level in Figure 7E. N = 6. Any two bars with different letters indicate that these two groups are statistically significantly different (*p*<0.05).


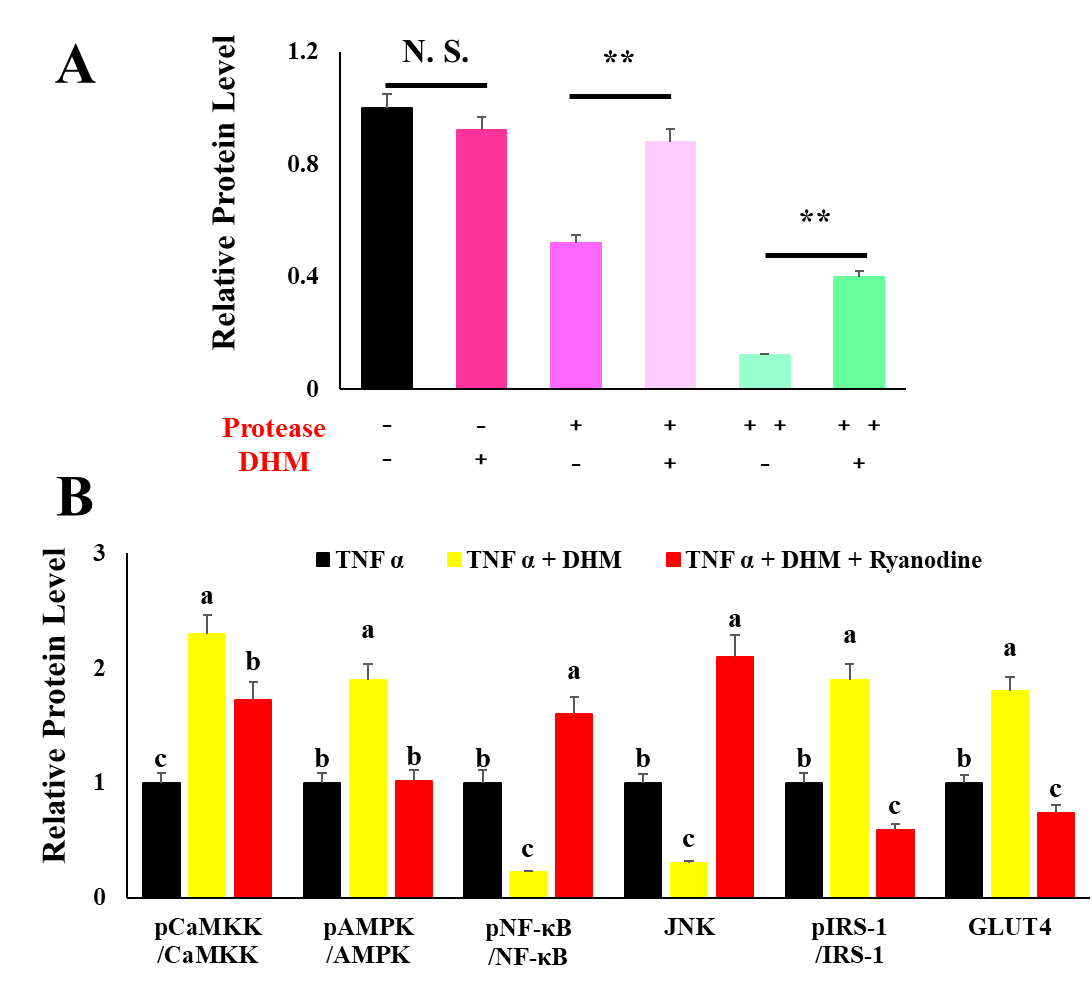


Figure S8. Statistical results related to Figure 8. A. Grayscale scanning statistics of PLC protein level in Figure 8B. B. Grayscale scanning statistics of inflammatory induced insulin resistance-related protein level in Figure 8D. N = 6. ** *p* < 0.01. Any two bars with different letters indicate that these two groups are statistically significantly different (*p*<0.05).
